# A Novel Real-Time PCR Assay of microRNAs Using S-Poly(T), a Specific Oligo(dT) Reverse Transcription Primer with Excellent Sensitivity and Specificity

**DOI:** 10.1371/journal.pone.0048536

**Published:** 2012-11-13

**Authors:** Kang Kang, Xiaoying Zhang, Hongtao Liu, Zhiwei Wang, Jiasheng Zhong, Zhenting Huang, Xiao Peng, Yan Zeng, Yuna Wang, Yi Yang, Jun Luo, Deming Gou

**Affiliations:** 1 College of Life Sciences, Shenzhen University, Shenzhen, Guangdong, People’s Republic of China; 2 College of Animal Science and Technology, Northwest A&F University, Yangling, Shaanxi, People’s Republic of China; 3 Department of Cardiovascular Surgery, Shenzhen Sun Yat-Sen Cardiovascular Hospital, Shenzhen, Guangdong, People’s Republic of China; 4 School of Medicine, Shenzhen University, Shenzhen, Guangdong, People’s Republic of China; National Institutes of Health, United States of America

## Abstract

**Background:**

MicroRNAs (miRNAs) are small, non-coding RNAs capable of postranscriptionally regulating gene expression. Accurate expression profiling is crucial for understanding the biological roles of miRNAs, and exploring them as biomarkers of diseases.

**Methodology/Principal Findings:**

A novel, highly sensitive, and reliable miRNA quantification approach,termed S-Poly(T) miRNA assay, is designed. In this assay, miRNAs are subjected to polyadenylation and reverse transcription with a S-Poly(T) primer that contains a universal reverse primer, a universal Taqman probe, an oligo(dT)_11_ sequence and six miRNA-specific bases. Individual miRNAs are then amplified by a specific forward primer and a universal reverse primer, and the PCR products are detected by a universal Taqman probe. The S-Poly(T) assay showed a minimum of 4-fold increase in sensitivity as compared with the stem-loop or poly(A)-based methods. A remarkable specificity in discriminating among miRNAs with high sequence similarity was also obtained with this approach. Using this method, we profiled miRNAs in human pulmonary arterial smooth muscle cells (HPASMC) and identified 9 differentially expressed miRNAs associated with hypoxia treatment. Due to its outstanding sensitivity, the number of circulating miRNAs from normal human serum was significantly expanded from 368 to 518.

**Conclusions/Significance:**

With excellent sensitivity, specificity, and high-throughput, the S-Poly(T) method provides a powerful tool for miRNAs quantification and identification of tissue- or disease-specific miRNA biomarkers.

## Introduction

MicroRNAs (miRNAs) are small, non-coding, single-stranded RNAs capable of negatively regulating gene expression [1]. Biogenesis of mature miRNAs (18–25 nt in length) occurs through a multi-step process that begins with the cleavage of the primary miRNA (pri-miRNA) by the endonuclease, Drosha, to produce a ∼70 nt hairpin precursor miRNA (pre-miRNA). After being exported to the cytoplasm by expotin 5, the pre-miRNA is further cleaved by the endonuclease, Dicer, to produce a mature miRNAs [2], [3]. As part of a multiprotein RNA-induced silencing complex (RISC), mature miRNAs guide the binding of RISC to the specific targets and promote their degradation and/or translational inhibition [4]. Increasing evidence has revealed that miRNAs play multiple regulatory roles in various biological processes, including embryo development [5], cell differentiation [6], proliferation [7], apoptosis [8], and many diseases [9], [10], [11], [12]. miRNAs have also been identified as biomarkers of various human diseases. Particularly, circulating miRNAs are being investigated as blood-based markers for cancer detection. [13], [14], [15]. Expression profiling of miRNAs in the specific cells, tissues or blood of interest have therefore become extremely important not only for understanding their fundamental roles but also for exploring them as novel biomarkers for diagnosis and prognosis of human diseases.

Over the past years, more than 30 different methods have been developed to measure miRNA expression, such as northern blot [16], microarray [17], [18], deep sequencing [19], [20] and real-time quantitative PCR (RT-qPCR) [21], [22], [23], [24]. Of these methods, qPCR is most sensitive and usually exploited to validate the data obtained from the high-throughput approaches. To date, there are two major qPCR-based strategies for miRNA quantification assay, namely the poly(A) method [25], [26], [27], [28] and the stem-loop method [21], [29], [30], [31]. The poly(A) method relies on polyadenylation of miRNAs by poly(A) polymerase, followed by cDNA synthesis with reverse transcriptase using a universal oligo(dT) primer. The poly(A) method enables simultaneous cDNA synthesis of all polyadenylated RNAs including mRNA, rRNA, tRNA, pri-, pre- and mature miRNAs followed by quantitative real-time qPCR with a miRNA-specific forward primer and a universal reverse primer. Although the poly(A) method is capable of assaying miRNA expression in a high-throughput manner, it is less specific due to the non-specific reverse transcription (RT). The stem-loop method uses stem-loop primers for the cDNA synthesis of miRNAs. Each stem-loop RT primer is unique for an individual miRNA, which gives a better specificity than the poly(A) method during the RT step. Nevertheless, as there are only few (usually six) bases that guide the binding of the 3′ end of the step-loop primer to the target miRNAs, the efficiency of the stem-loop method is relatively lower even with a pulse RT reaction [30]. Additionally, the stem-loop method requires individual miRNA-specific hydrolytic Taqman probes, making them very costly for a high-throughput miRNA expression profiling [32].

In this paper, we described a novel qPCR-based approach for miRNA expression analysis, termed S-Poly(T) miRNA assay. This method utilizes a S-Poly(T) primer that includes an oligo(dT)_11_ sequence and several miRNA-specific bases, permitting higher RT efficiency, and thus better sensitivity and specificity than the poly(A) and stem-loop methods.

## Materials and Methods

### Probe and Primers

The universal Taqman probe and all the primers (Table S1, S2, S3, S4, S5) are purchased from Integrated DNA Technologies (IDT).

### Cell Culture

Human HEK293 cells were purchased from American Type Culture Collection (ATCC, Manassas, VA) and maintained in Dulbecco's modified Eagle's medium (DMEM) supplemented with 10% fetal bovine serum (FBS) at 37°C and 5% CO_2_ humidified incubator. Primary human pulmonary arterial smooth muscle cells (HPASMC) from normal subjects were purchased from Lonza (Walkersville, MD) and cultured in SmGM-2 smooth muscle cell growth medium consisting of smooth muscle cell basal medium, 5% FBS, 0.5 ng/ml human recombinant epidermal growth factor, 2 ng/ml human recombinant fibroblast growth factor, 5 μg/ml insulin, and 50 μg/ml gentamicin. Hypoxia treatment of HPASMC at passage 8 was done in a special hypoxia incubator (model 3131; Thermo Fisher Scientific) infused with a gas mixture of 5% CO_2_, balance nitrogen to obtain ∼3% O_2_ concentration in the incubator. The HPASMC cultured under the normoxia condition was used as a control. Both hypoxia and normoxia treatments were conducted in triplicate.

### Overexpression of Human miRNAs with Lentivirus Infection

To construct miRNA overexpression vectors, eight pri-miRNAs in the let-7 family were PCR-amplified from human genomic DNA (Promega) with each specific primer set (Table S4). After the double digestion with XhoI/EcoRI (or BsaI), the PCR products were inserted into the downstream of the EGFP stop codon in pLVX/CMV-EGFP vector as we described before [33]. All constructs were confirmed by DNA sequencing. High-titer lentiviruses were packaged in 293T cells by co-transfecting a lentiviral vector and Lenti-X HT packaging plasmids (Clontech) with jetPEI® DNA Transfection Reagent (Polyplus Transfection, New York, NY). Same amounts of lentiviruses were used to infect HEK293 cells in the presence of Polybrene (4 µg/ml).

### Serum Collection

Human serum samples were collected from 40 healthy participants at the Sun Yat-Sen Cardiovascular Hospital (Shenzhen, China). The blood samples were allowed to stand at room temperature for 1 h and then centrifuged at 3,000 × g for 10 min at 4°C. The supernatant were stored at −80°C. This study was approved by the Institutional Ethics Committee at the Shenzhen Sun Yat-Sen Cardiovascular Hospital (Shenzhen, China), and informed written consents were obtained from all subjects.

### RNA Extraction

Total RNA from cultured cells was isolated using the RNAiso Plus (TaKaRa) according to the manufacturer’s protocol. Serum RNA was extracted using the mirVana PARIS kit (Ambion) based on the manufacturer’s instruction. Briefly, 500 µl serum of each sample was mixed with an equal volume of 2 × Denaturing Solution, and total RNA was eluted in 100 µl RNase-free water.

### Polyadenylation and Reverse transcription

For the S-Poly(T) method, total RNA from cultured cells was polyadenylated with Poly(A) Polymerase Tailing Kit (Epicentre). Briefly, a 10 µl reaction including 1 µg total RNA, 1 µl of 10 × reaction buffer, 1 µl of 10 mM ATP and 1 unit of Poly(A) polymerase was incubated at 37°C for 30 min, followed by enzyme inactivation at 65°C for 5 min. After polyadenylation, reverse transcription was performed in a 10 µl reaction containing 1 µl of the polyadenylation reaction product, 1 µl of 0.5 µM RT primer, 0.5 µl of 10 mM dNTP, 1 µl of MMLV HP RT 10 × reaction buffer, and 50 units of MMLV High Performance Reverse Transcriptase (Epicentre). The reaction was incubated at 42°C for 60 min, and then terminated by heating at 85°C for 5 min. For serum RNA sample, as the yield of RNA from serum was below the limit of accurate quantitation by spectrophotometry, a fixed volume of 7.75 µl eluted serum RNA, rather than a fixed mass of RNA, was added in a 10 µl polyadenylation reaction. For the Poly(A) method, the procedure was the same as that of the S-Poly(T) method except that 1 µl of 1 µM universal RT primer (Table S2) was used in a 10 µl RT reaction. For the stem-loop method, the polyadenylation step was omitted. RT reaction containing 100 ng of total RNA in a 10 µl reaction volume was carried out in the same manner as the S-Poly(T) method except that 1 µl of 0.5 µM stem-loop RT primer (Table S3) was used. For the comparison experiment, the expression of hsa-miR-140-5p was measured with the TaqMan microRNA assay kit (Applied Biosystems) according to the manufacturer’s instructions. The stem-loop RT reaction was performed at 16°C for 30 min, followed by 42°C for 30 min, and 85°C for 5 min. No-template control (NTC) and no-reverse transcriptase control (-RT) were included in all RT reactions.

### Real-time PCR

For the S-Poly(T) method, the RT products can be amplified and detected using either a universal Taqman probe or SYBR Green I. When using a universal Taqman probe, a 20 µl PCR reaction contains 0.5 µl of RT products, 4 µl of 5× Colorless GoTaq® Flexi Buffer, 0.5 unit of GoTaq® Hot Start Polymerase (Promega), 1.5 mM MgCl_2_, 0.2 mM dNTP, 0.2 mM forward primer, 0.2 mM universal reverse primer, 0.25 mM universal Taqman probe, and 5 µM ROX reference dye. The PCR reaction was performed at 95°C for 2 min, followed by 40 cycles of 95°C for 10 s and 60°C for 30 s.

To compare the sensitivity among S-Poly(T), poly(A) and stem-loop methods, an equal amount of cDNAs was amplified with the same pair of PCR primers for the corresponding miRNA and detected with SYBR Green I. For using SYBR Green I, a 20 µl reaction includes 0.5 µl of RT products, 10 µl of SYBR® Premix Ex Taq™ (TaKaRa), 0.2 mM forward primer, 0.2 mM universal reverse primer, and 5 µM ROX reference dye. The reaction was performed at 95°C for 30 s, 40 cycles of 95°C for 5 s, and 60°C for 30 s followed by a melting curve analysis. To further compare the S-Poly(T) and stem-loop methods with Taqman probes, hsa-miR-140-5p was assayed using either S-Poly(T) procedure described above or TaqMan® microRNA assay kit (Applied Biosystems) according to the manufacturer’s instructions. All reactions were run in triplicate and the qPCR reactions were carried out on ABI PRISM 7300 thermal cycler. Increased sensitivity of the S-Poly(T) method compared with poly(A) or stem-loop method was calculated using 2^∧^-ΔCt.

### Cellular and Serum miRNA Profiling

A total of 1,080 human mature miRNAs based on the miRBase Release 16 were selected for a high-throughput miRNA profiling assay. A simultaneous synthesis of cDNA for 360 miRNAs and one internal control SNORD47 was performed in a single RT reaction and the final concentration of each RT primer in a RT reaction was 1.67 nM. For HPASMC miRNA profiling assay, a total of 4 µg RNA sample from 3 biological replicates undergoing 24 h hypoxia (3% O_2_) or normoxia treatment was used for polyadenylation and subsequent reverse transcription. A 20 µl qPCR of each miRNA was performed in triplicate containing 0.1 µl of RT products corresponding to 1 ng of total RNA. The expression of miRNAs was normalized to the small nucleolar RNA, SNORD47. For serum miRNA profiling, 40 healthy serum RNA samples were mixed equally and 1 µl of RT product was included in a 20 µl qPCR reaction. After reverse transcription, miRNAs were assayed in a 384-well plate on a Bio-Rad CFX384 real-time system. Data analysis was performed by the comparative threshold cycle (Ct) method. Those data with a Ct value >35 were omitted. The miRNAs with fold change ≥2 were selected for validation in each individual sample. Statistical analysis was performed with GraphPad Prism 5 using a two-tailed Student’s t-test. A P-value <0.05 was considered statistically significant.

### LNA-based miRNA Microarray

Normoxic and hypoxic HPASMC total RNA samples (4 µg) were labeled using the miRCURY LNA Array Labeling Kit according to the manufacturer’s instructions (Exiqon, Vedbaek, Denmark). The hybridization was performed according to the miRCURY LNA array manual. Following hybridization, the slides were washed by Wash buffer kit (Exiqon, Vedbaek, Denmark), dried and scanned. Signals were normalized using global Lowess, and the results were calculated as Hy3/Hy5 ratio between normoxic and hypoxic HPASMC samples.

## Results

### Design of a Novel Real-time PCR Method for Quantification of Mature miRNAs

**Figure 1:**
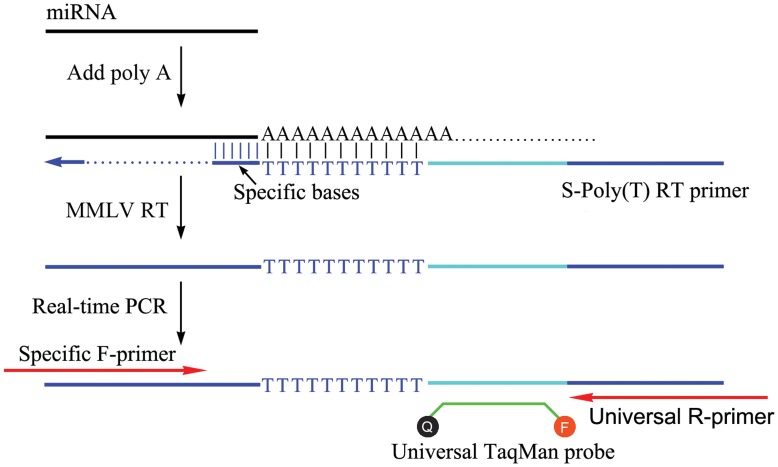
Schematic representation of the S-Poly(T) method for miRNA quantification. miRNAs are subjected to polyadenylation and subsequent reverse transcription with miRNA-specific S-Poly(T) RT primers. The S-Poly(T) RT primers consist of four segments, in the 5′ to 3′ direction, a universal reverse primer sequence for PCR, a universal Taqman probe sequence and an oligo(dT)_11_, followed by 5∼7 specific bases that are complementary to the 3′ end of a particular mature miRNA. PCR products were generated with a miRNA-specific forward primer and a universal reverse primer and detected by a universal Taqman probe.

A novel miRNA qPCR method was developed using an elaborately designed RT primer, S-Poly(T). The S-Poly(T) primer is composed of four segments, in the 5′ to 3′ direction, a universal reverse primer sequence for PCR (5′- CAGTGCAGGGTCCGAGGT-3′), a universal Taqman probe sequence (56-FAM/CAGAGCCAC/ZEN/CTGGGCAATTT/3IABkFQ) and an oligo(dT)_11_ sequence, followed by 5∼7 (usually six) specific bases that are complementary to the 3′ end of a particular miRNA (Figure 1). The unique universal reverse primer sequence containing 67% of GC was designed not to share significant similarity with any sequences in the GenBank database. For miRNA quantification, a poly(A) tail is added to the 3′ end of each mature miRNA by the poly(A) polymerase. Because of the presence of mature miRNA-specific sequence in the S-Poly(T) primer, only polyadenylated mature miRNAs are then reverse transcribed to cDNA. Individual miRNA is amplified with a specific forward primer and a universal reverse primer. PCR products are detected by a universal Taqman probe (Figure 1).

### Dynamic Range and Sensitivity of the S-Poly(T) miRNA Assay

**Figure 2:**
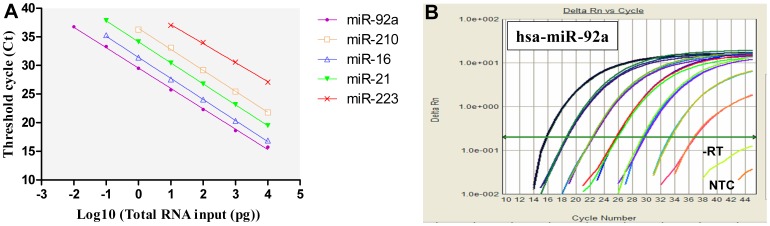
Dynamic range and sensitivity of the S-Poly(T) method used in miRNA quantification. A: Correlation of total RNA input to the threshold cycle (Ct) value. Five miRNAs including hsa-miR-92a, hsa-miR-210, hsa-miR-16, hsa-miR-21 and hsa-miR-223 were assayed by the S-Poly(T) method with a series of 10-fold diluted total RNAs prepared from HEK293 cells. The amount of total RNA in each qPCR reaction was ranged from 10 ng to 0.01 pg. The amplification efficiency was 96% (miR-92a), 95% (miR-210), 91% (miR-16), 90% (miR-90), and 101% (miR-223), respectively, and the correlation coefficients (R^2^) for each miRNAs was >0.99. B: Amplification plot of hsa-miR-92a over seven orders of magnitude. NTC: no-template control; -RT: no-reverse transcriptase control. All PCR reactions were performed in triplicate.

To evaluate the S-Poly(T) method, we focused on three critical criteria: dynamic range, sensitivity and specificity. To determine the dynamic range and sensitivity of the S-Poly(T) miRNA assay, a series of 10-fold diluted total RNAs from HEK293 cells, ranging from 1 µg to 1 pg, were tailed with poly(A) polymerase, and then reverse transcribed to cDNA with five mixed RT primers corresponding to hsa-miR-92a, hsa-miR-210, hsa-miR-16, hsa-miR-21, and hsa-miR-223. One microliter of cDNA samples corresponding to 10 ng to 0.01 pg of initial total RNAs was used as a template to evaluate the expression of each of the five miRNAs with a miRNA-specific forward primer and a universal reverse primer. The result demonstrated an excellent linearity between the total RNA input and the threshold cycle (Ct) value for all tested miRNAs (Figure 2A), with miR-92a showing the broadest dynamic range of seven orders of magnitude (Figure 2B). The limit of detection (LOD) for miR-92a was 0.01 pg total RNA in a qPCR reaction. For miR-16 and miR-21, the LOD was 0.1 pg of total RNA. The LOD for miR-210 and miR-223 (a low abundant miRNA in HEK293 cells) in a qPCR reaction were 1.0 pg and 10.0 pg, respectively. There was no significant detection signal in no-template control (NTC) and no-reverse transcriptase (-RT) control.

**Figure 3:**
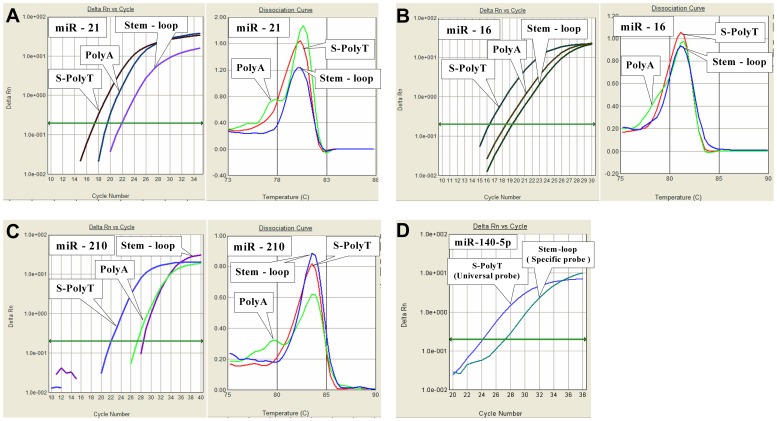
Comparison of the sensitivity of three different miRNA qPCR assays. Total RNAs were reverse transcribed into cDNA using S-Poly(T) RT primer, oligo(dT) RT primer and Stem-loop RT primer, respectively. Hsa-miR-21 (A), hsa-miR-16 (B) and has-miR-210 (C) were amplified and detected with SYBR Green I followed by melting curve analysis. Hsa-miR-140-5p was reverse transcribed into cDNA using the S-Poly(T) RT primer and the RT primer from TaqMan® microRNA assays kit (Applied Biosystems). The cDNAs were then amplified and detected with either a universal Taqman probe (S-Poly(T) method) or a specific Taqman probe (stem-loop method). The threshold was set at 0.2 on PRISM 7300 Real-time PCR system (Applied Biosystems). All PCR reactions were run in triplicate.

The broad dynamic range and excellent sensitivity are mainly dependent on the employment of a novel S-Poly(T) RT primer. Generally, a S-Poly(T) primer contains 17 bases (11 dT and 6 specific bases) annealing sequences that are long enough to anneal to the polyadenylated miRNAs specifically at elevated temperatures (42∼50°C) and thus provides a better RT efficiency and subsequent higher sensitivity in miRNA quantification than the conventional poly(A) or stem-loop approach. An experiment comparing three different qPCR methods, such as S-Poly(T), poly(A) and stem-loop methods in miRNA assay was carried out in this study. Three miRNAs (miR-21, miR-16 and miR-210) with different expression levels in the HEK293 cells were reverse transcribed into cDNAs with S-Poly(T), oligo(dT) or stem-loop RT primers, respectively. PCR reactions running on a same plate were assessed by the incorporation of SYBR Green I simultaneously. It is very clear that the S-Poly(T) method produced the smallest Ct values for all tested miRNAs, followed by the poly(A) and stem-loop methods (Figure 3A∼C). For miR-21, the Ct value with the S-Poly(T) assay was decreased by 2.11 and 4.60 when compared to the poly(A) and stem-loop methods, meaning that the S-Poly(T) method was 4.3 and 24.3 times more sensitive than the poly(A) and stem-loop method for miR-21 detection (Table 1). Similarly, the sensitivity with the S-Poly(T) method was increased 4.6/8.3 times and 35.5/91.1 time for miR-16 and miR-210, respectively, in comparison with the poly(A) and stem-loop methods. These results demonstrated that the S-Poly(T) method is much more sensitive than other two methods. We also noticed that the Ct values from the poly(A) method were always smaller than that that from the stem-loop method, which seems that the poly(A) method is more sensitive than the stem-loop method. However, the poly(A) method gave more than one peaks according to the melting curve analysis, indicating that the smaller Ct values generated with this method may be, to some extent, due to the non-specific amplification signal. In other words, the stem-loop method is more specific than the poly(A) method in miRNA assay.

**Table 1:** The Ct values of miRNA qPCR assays using three different methods.

miRNAs	Ct values*			Increased sensitivity (2^∧^-ΔCt)	
	S-Poly(T)	poly(A)	stem-loop	S-Poly(T)/poly(A)	S-Poly(T)/stem-loop
miR-21	17.29±0.09	19.40±0.13	21.89±0.15	4.3	24.3
miR-16	16.26±0.08	18.47±0.17	19.32±0.13	4.6	8.3
miR-210	22.09±0.12	27.24±0.09	28.60±0.12	35.5	91.1
miR-140-5p	24.13±0.11	NA	27.21±0.14	NA	8.5

* : miR-21, miR-16 and miR-210 were assayed with SYBR Green I, while miR-140-5p was assayed with universal (S-Poly(T)) or specific (stem-loop) Taqman probe.

Although the results from SYBR Green I dye are acceptable, we prefer to use the universal Taqman probe for qPCR detection because it eliminates the non-specific amplifications. Another reason is that stem-loop method with the specific Taqman probe has been widely used [21]. So, we compared the sensitivity between our S-Poly(T) method with a universal Taqman probe and the ABI-provided miRNA assay kit with specific Taqman probe. It showed that S-Poly(T) method with a universal Taqman probe was 8.5-fold more sensitive than stem-loop method with a specific Taqman probe in miR-140-5p assay (Figure 3D, Table 1), convincingly demonstrating the superior sensitivity of the S-Poly(T) method.

### Specificity of the S-Poly(T) miRNA Assay

**Figure 4:**
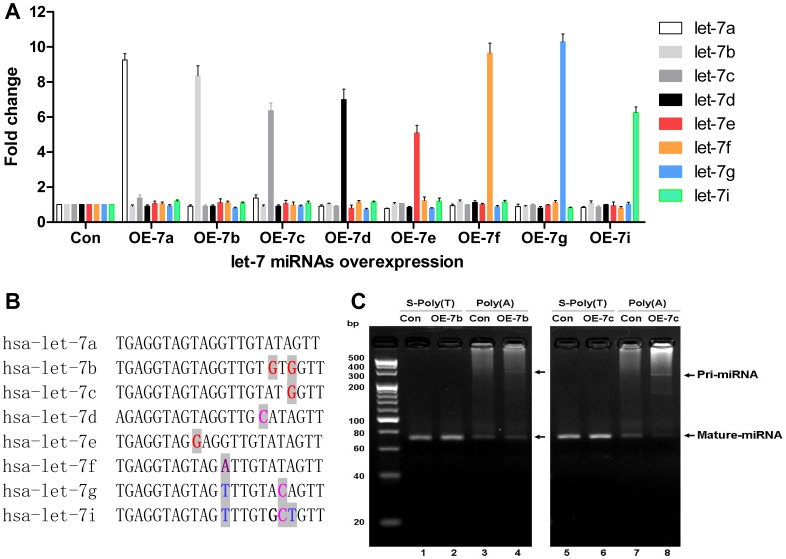
Specificity of the S-Poly(T) method in human let-7 family miRNAs assay. A: Eight hsa-let-7 miRNAs (let-7a, let-7b, let-7c, let-7d, let-7e, let-7f, let-7g, let-7i) were individually over-expressed in HEK293 cells by a lentiviral vector. The expression levels of the eight hsa-let-7 miRNAs were compared between the control (transduced with blank lentiviral vector) and the hsa-let-7 miRNAs-overexpressing HEK293 cells. The expression level of each hsa-let-7 miRNA in the hsa-let-7 miRNAs-overexpressing HEK293 cells (OE-7a∼OE-7i) was shown as the fold change compared with that in the control (Con). B: sequence alignment of eight hsa-let-7 miRNAs. Non-conserved nucleotides are shaded. C: discrimination of mature miRNA from pri- and/or pre- miRNA by the S-Poly(T) and Poly(A) methods. The total RNA overexpressing hsa-let-7b and hsa-let-7c were reverse transcribed to cDNA by the S-Poly(T) and Poly(A) methods, respectively. The PCR products were run on 4% agarose gel in sodium borate (SB) buffer. The arrows indicate the amplification products of pri- and mature miRNAs.

To evaluate the specificity of the S-Poly(T) method, we determined the ability of the S-Poly(T) method to discriminate among miRNAs with high sequence homology. Eight of miRNAs in the hsa-let-7 family (let-7a, let-7b, let-7c, let-7d, let-7e, let-7f, let-7g, and let-7i) (Figure 4B) were individually over-expressed in HEK293 cells by transducing lentivirus-based pri-miRNA vectors. The expression of each let-7 miRNA in the control HEK293 cells (transduced with pLVX-CMV-EGFP lentiviral vector) and the let-7-overexpressing HEK293 cells were evaluated with the S-Poly(T) method. Results showed that all of the eight let-7 miRNAs were successfully over-expressed in HEK293 cells respectively, with the fold changes ranging from 5.1 to 10.3 (Figure 4B). However, we did not see a significant “cross talk” detection of other let-7 family members, indicating that the S-Poly(T) method is specific to distinguish the miRNA homologs, with even a single base difference.

Mature miRNAs, rather than pri- and/or pre-miRNAs, play an essential role in the post-transcriptional regulation of gene expression. Hence, a precise evaluation of the mature miRNA expression is of great importance. In each S-Poly(T) RT primer, the 3′ end of six bases specifically complementary to corresponding miRNA(s) are linked to the oligo(dT)_11_ sequence. Theoretically, only the polyadenylated mature miRNA could be recognized by the corresponding S-Poly(T) primer in the RT reaction (≥42°C), conferring the ability to distinguish the mature miRNAs from the pri- or pre-form. To test this, the samples overexpressing hsa-let-7b and hsa-let-7c were assayed with the S-Poly(T) and poly(A) methods, respectively. The PCR amplification products were visualized on 4% sodium borate (SB)-buffered agarose gel electrophoresis. The results showed that, neither S-Poly(T) nor poly(A) method can detect any pri- or pre-miRNAs in the control samples (Figure 4C, lane 1, 3, 5 and 7). However, in hsa-let-7b or hsa-let-7c over-expressing HEK293 cells, both the mature miRNAs (∼70 bp weak bands) and pri-miRNAs (∼300 bp sharp bands) can be amplified by the poly(A) method (Figure 4C, lane 4 and 8), suggesting that the poly(A) method cannot distinguish well between mature miRNAs and pri-miRNAs. Moreover, there are many non-specific products appearing on the gel when using the poly(A) method (Figure 4C, lane 4, 8), confirming the non-specific signal shown by the melting curve analysis of the poly(A) method (Figure 3). However, the S-Poly(T) method produced only the products corresponding to the mature miRNAs (Figure 4C, lane 2, 6), indicating that the S-Poly(T) method can specifically discriminate mature miRNA from pri- or pre-miRNA.

### High-throughput miRNA Profiling in Hypoxia-treated HPASMC

**Figure 5:**
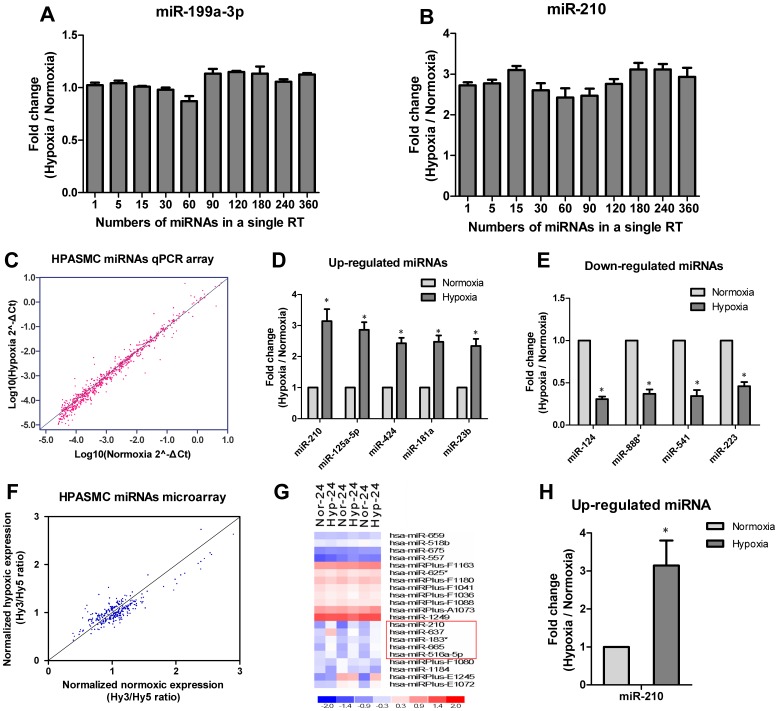
MiRNAs profiling between hypoxic and normoxic HPASMC. A, B: The accuracy for quantification of miR-199a-3p (A) and miR-210 (B) was evaluated in a high-throughput RT reaction. The number of pooled RT primers in a single RT reaction ranged from 1∼360. C: Scatter plot of relative expression level of 721 miRNAs determined by S-Poly(T) qPCR. The relative expression of miRNA was calculated as 2^∧^-ΔCt and presented in the logarithmic scale. The data on the line across both X and Y axes represented the constitutively expressed miRNAs. D, E: Significantly up-regulated and down-regulated miRNAs. The expression of miRNAs was shown as fold change between hypoxia and normoxia. F: Scatter plot of the relative expression level of 246 miRNAs detected by LNA-based miRNA microarray. The relative expression of miRNA was calculated as Hy3/Hy5 ratio and the data on the line across both X and Y axes represented the constitutively expressed miRNAs. G: Heat map showing the expression of miRNAs that are differentially expressed in hypoxic HPASMC compared with normoxic HPASMC. Red color denotes induction and blue color denotes suppression. H: Significantly up-regulated miRNAs verified by real-time PCR. The expression of miRNAs was shown as fold change between hypoxia and normoxia. Each bar represents mean ± SD. Statistical significance was determined using two-tailed Student’s t-test. * P<0.05.

High-throughput miRNAs expression profiling has become a popular discovery tool to identify miRNA functions in many cellular processes. One application of the S-Poly(T) technique described here is quantitative analysis of miRNA expression on a large scale. Pulmonary arterial hypertension (PAH) is an extremely severe, life-threatening disorder characterized by abnormal vascular smooth muscle cell proliferation [34]. Recently, several groups have reported that miRNAs play an important role in the pathogenesis of PAH [9], [33], [35]. Since hypoxia is one of the important stimulus for HPASMC proliferation and PAH, we evaluated the differential expression of miRNAs in response to hypoxia using the S-Poly(T) method. To test if a simultaneous cDNA synthesis can be used for profiling of multiple miRNAs, we examined the expression of miR-199a-3p and miR-210 using the mixed cDNA samples prepared from the pooled RT primers (including 1, 5, 15, 30, 60, 90, 120, 180, 240, and 360 pre-mixed RT primers) in a single RT reaction. As Figure 5A & 5B illustrated, both miR-199a-3p and miR-210 showed almost identical fold changes between normoxia and hypoxia samples when tested with the RT primers that were differently pooled, suggesting an accepted accuracy of the S-Poly(T) method with pooled RT primers.

Subsequently, 1,080 miRNAs on the base of miRBase 16.0 were divided into 3 groups of 360 miRNAs for high-throughput miRNAs profiling at 384-well plates. The RT reactions were performed with the pre-mixed RT primers including one internal control RT primer for SNORD47, using the polyadenylated HPASMC total RNAs as templates. Out of the 1,080 miRNAs tested, 276 miRNAs with no amplification signals or Ct values >35 in either of the normoxia or hypoxia samples were excluded in the data analysis step. The specificity of the remaining miRNA was then evaluated by analyzing the PCR products on 4% SB-buffered agarose gel electrophoresis. Eighty-three miRNAs were further excluded because of non-specific amplifications. The expressions of 721 specific and significant miRNAs in both hypoxia and normoxia HPASMC were calculated (Table S5) and showed in a scatter plot (Figure 5C). We further picked up 64 miRNAs with significant changes for verification in individual samples. miR-210, miR-125a-5p, miR-424, miR-181a and miR-23b were most significantly up-regulated in hypoxia-treated HPASMC (Figure 5D), whereas miR-124, miR-888*, miR-541 and miR-223 were the most down-regulated miRNAs (Figure 5E).

To allow a direct comparison between the S-Poly(T)-based miRNA qPCR array and miRNA microarray, the same RNA samples used in qPCR array were also applied for miRNA expression profiling using LNA-based miRNA microarray (Exiqon, Vedbaek, Denmark). Among 1,265 human miRNAs included in microarray, a total of 286 miRNAs can be detected and most of them did not show any significant change in hypoxia (Figure 5F). Only five miRNAs (miR-210, miR-665, miR-637, miR-183* and miR-516a-5p) showed >1.5-fold induction (Figure 5G). However, miR-210 was the only validated miRNA induced by hypoxia significantly (Figure 5H), as miR-665, miR-637, miR-183* and miR-516a-5p all resulted in non-specific amplification with the S-Poly(T) method (data not shown). We also assayed miR-665, miR-637, miR-183* and miR-516a-5p using the miRCURY LNA microRNA PCR System (Exiqon), obtaining the same results as the S-Poly(T) method. Obviously, the S-Poly(T)-based miRNA qPCR array is much more sensitive and accurate than the LNA-based miRNA microarray.

### Circulating miRNA Profiling in Human Serum

**Figure 6:**
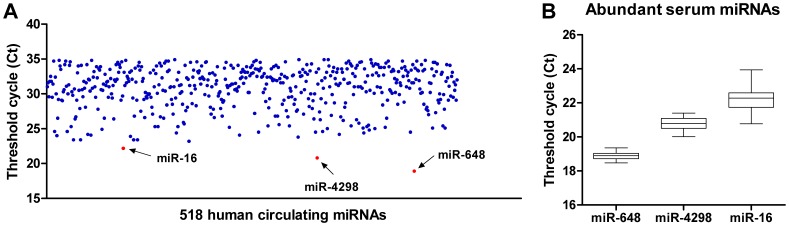
Expression profiling of human serum miRNAs. A: Pooled total RNAs from 40 healthy human serum samples were used to assay human 1,080 miRNAs in the miRBase 16.0. A scatter plot of 518 human serum miRNAs with Ct <35 assayed by S-Poly(T) method was shown. Arrows indicate the most abundant miRNAs. B: Box-whisker plots of the three most abundant miRNAs, namely miR-648, miR-4298 and miR-16 in each healthy serum sample (n = 40). The boxes indicate the 75th and 25th percentiles. The whiskers denote the lowest and highest values. The lines inside the boxes indicate the median values.

Recently, miRNAs were detected in the circulation, including serum and plasma [36], implying a great potential of using circulating miRNAs as non-invasive biomarkers for human diseases [37], [38], [39]. However, a minute amount of miRNAs in serum greatly challenges the method used for their detection. The repertoire of serum miRNAs still remains unclear. Thus, we pooled 40 healthy serum samples and performed a miRNA profiling assay using the S-Poly(T) approach. As a result, 518 miRNAs were detectable (Ct <35) showing specific amplifications with predicted sizes of PCR products (Figure 6A and Table S6). Among them, miR-648, miR-4298, and miR-16 were among the most abundant circulating miRNAs, with a Ct value of 18.9, 20.8, and 22.2, respectively. Although miR-16 [40], [41] and miR-648 [42] are known to be present at high levels in serum, this is the first report that miR-4298 also exists abundantly in the circulation. Further assaying these three miRNAs in individual serum sample showed that miR-648 and miR-4298 had relatively narrow expression ranges, with Ct values ranging from 18.5 to 19.4, and 20.0 to 21.4, respectively (Figure 6B). In contrast, miR-16 displayed a wide expression range of 20.8 ∼ 23.9 in Ct value.

## Discussion

We have developed a novel approach for miRNA expression profiling using a uniquely designed S-Poly(T) RT primer. With presence of an oligo(dT) and miRNA-specific sequences, the S-Poly(T) RT primer only anneals to and reverse transcribes a corresponding polyadenylated mature miRNA, which provides higher RT efficiency, and thus better sensitivity and specificity in miRNA qPCR assay.

Currently, the two commonly used real-time PCR approaches in miRNA assessment are the poly(A) and stem-loop methods. The RT primer of the poly(A) method is composed of an oligo(dT) sequence followed by 2 degenerate nucleotides. With this RT primer, the poly(A) method enables reverse transcription of all polyadenylated RNAs including mRNA, pri-, pre- and mature miRNAs. Such a high-throughput manner of RT reaction provides great simplicity in miRNA assays. However, the indiscriminate RT reaction with poly(A) method largely reduces the specificity for miRNA profiling. Moreover, a decrease in specificity may further impair the sensitivity, due to the competition of the RT primer to bind to the non-specific templates. In contrast, the stem-loop RT primer that consists of several miRNA-specific bases and a stem-loop secondary structure, may allow better specificity than the poly(A) method. However, the anchoring strength of the stem-loop primer to the miRNA molecules may not be strong enough, because of the availability of merely several (usually six) bases complementary to the miRNA template. Accordingly, the RT efficiency may be limited, leading to a decrease in the sensitivity, which was confirmed in our studies showing the larger Ct values in analysis of several miRNA (Figure 3).

Distinctively, the S-Poly(T) RT primer combines the advantages of both the stem-loop and poly(A) methods, i.e., the specificity of the stem-loop RT primer and the relatively high anchoring strength of the oligo(dT)_11_ of the poly(A) method. In general, there are 17 nucleotides (11 dTs and 6 specific bases) at the 3′ end of each S-Poly(T) RT primer that can anneal to the polyadenylated target miRNA, providing both higher binding strength and thermodynamic stability between the RT primer and miRNA template. Consequently, the RT efficiency is greatly enhanced, which further improves both the sensitivity and specificity in the miRNA assay. We showed in this work that hsa-miR-92a was successfully detected in as little as 0.01 pg of total RNA. Given that total RNA in a single cell is approximately 15 pg [21], the S-Poly(T) method is thus believed to be sensitive enough to detect the miRNA expression in a single cell. When compared with the poly(A) and stem-loop methods, the S-Poly(T) method showed at least 4-fold increase in sensitivity in miRNA quantification (Figure 3).

The S-Poly(T) method with enhanced sensitivity will prove invaluable for quantification of a small amount of miRNAs in biological samples like serum, plasma or other body fluid. Circulating miRNAs are highly stable and correlated with physiological and pathological events [14], [15], making them promising biomarkers in disease diagnosis and prognosis. However, a limited miRNA amount in serum/plasma demands a sensitive assay. It is challenging to analyze the expression of serum miRNA using microarray, since a large amount of RNA sample is needed. Stem-loop qPCR has been used for the detection of circulating miRNA [37], [38], [39], [43]. However, low abundant serum miRNAs potentially requires a PCR pre-amplification step [39], [41], [43], which might increase bias in the subsequent miRNA quantification [14]. In contrary, the S-Poly(T) method is sufficiently sensitive for the circulating miRNA analysis, with no need of a pre-amplification step. We successfully detected 518 miRNAs from human serum with the S-Poly(T) qPCR method. To the best of our knowledge, only 368 miRNAs was detected among 667 miRNAs that were examined in the serum samples of the prostate cancer patients [39]. However, as the author did not list the 368 miRNAs in detail, we do not know if they are among the 518 miRNAs we identified. Anyhow, our work has greatly expanded the number of circulating miRNAs in the healthy serum, facilitating further studies on their functions in the circulation.

The specificity of the S-Poly(T) method was confirmed by its capability of discriminating among miRNAs with high sequence similarity. Let-7 family miRNAs were often chosen for specificity studies [21], [44]. However, many used the synthetic let-7 miRNAs, the genesis of which is different from the native mature miRNAs [21], [44]. In this study, we produced mature let-7 miRNAs in excess by transducing HEK293 cells with lentiviral vectors expressing the primary sequence of let-7 miRNAs. This allowed the accumulation of pri-, pre-, and mature forms of miRNAs in total RNA samples, offering a more reliable way of specificity assay than using synthetic miRNAs. We showed that S-Poly(T) method is capable of distinguishing the mature miRNAs from pri- and pre-miRNAs.

Using the S-Poly(T) method, we profiled miRNA expression in human pulmonary arterial smooth muscle cells (HPASMC), identifying nine differentially expressed miRNAs related with hypoxia treatment. Consistent with previous reports [45], [46], miR-210 demonstrated the most pronounced increase induced by hypoxia in HPASMC. Hypoxia-induced miR-424 expression was also observed in human endothelial cells [47]. Among the down-regulated miRNAs, miR-223 expression decreased in hypoxia-treated mouse lungs and such a down-regulation contributed to the development of hypoxia-induced PH and right ventricular failure [48]. Additionally, miR-124 was reported to be capable of inhibiting the proliferation of gastric cancer cells [49]. Down-regulation of miR-124 in hypoxia-treated HPASMC implies its potential regulatory effect on the HPASMC proliferation. Taken together, we demonstrated that the S-Poly(T) method is effective for screening differential miRNAs in a high-throughput profiling assay.

By far, the stem-loop method is the most popular approach in the high-throughput miRNA profiling assay. However, the stem-loop method utilizes miRNA-specific Taqman probes, the high cost of which might prevent it from being extensively used. Instead, the S-Poly(T) method employs a universal Taqman probe for all miRNAs, making it more cost-effective. In fact, the use of a universal hydrolytic probe was reported earlier in Locked Nucleic Acids (LNA)-spiked Universal Probe Library (UPL)-based miRNA quantification [26], [32]. Although LNA-modified probes can change the melting temperatures of oligonucleotides, the LNA technique might decrease the qPCR amplification efficiency [27].

In summary, the unique S-Poly(T) method has superior sensitivity, specificity, and efficiency in miRNA quantification assay, providing a powerful tool for identifying tissue-specific and disease-specific miRNA biomarkers.

## Supporting Information

Table S1 Primers and probe sequences for S-PolyT method.
(XLS)

Table S2 Primers sequences for polyA method.
(XLS)

Table S3 Primers sequences for stem-loop method.
(XLS)

Table S4 Primers for miRNA vector construction.
(XLS)

Table S5 MiRNAs profiling between hypoxic and normoxic HPASMC.
(XLS)

Table S6 Human serum miRNAs profiling.
(XLS)
